# Effect of Botulinum Toxin A on Scar Healing after Thyroidectomy: A Prospective Double-blind Randomized Controlled Trial

**DOI:** 10.3390/jcm9030868

**Published:** 2020-03-21

**Authors:** Dong Sik Bae, Do Hoon Koo, Ji Eun Kim, Jae-mahn Cho, Jun-Ook Park

**Affiliations:** 1 Department of Surgery, Haeundae Paik Hospital, Inje University College of Medicine, Busan 48108, Korea; md.ds.bae@gmail.com (D.S.B.); dohoon.koo.md@gmail.com (D.H.K.); 2 Department of Dermatology, CNP Skin Laser Clinic, Seoul 06267, Korea; jee0406@naver.com; 3 Department of Otolaryngology-Head and Neck Surgery, Haeundae Paik Hospital, Inje University College of Medicine, Busan 48108, Korea; cjm9803@nate.com; 4 Department of Otolaryngology-Head and Neck Surgery, College of Medicine, The Catholic University of Korea, Seoul 03312, Korea

**Keywords:** botulinum toxin type A, wound healing, hypertrophic scar, Thyroid, Thyroidectomy

## Abstract

The persistence of neck scarring is a common concern among patients undergoing thyroidectomy. Botulinum toxin A (BTA (Botox)) has been shown to suppress scar enlargement at the incision site. The objective of this study was to evaluate the effect of intraoperative Botox administration on neck scarring after thyroidectomy. A prospective double-blind randomized clinical trial was performed in patients undergoing conventional thyroidectomy. Forty patients were randomly allocated to a Botox or a control group (both, *n* = 20). The wound was closed after injection into the platysma muscle of 50 U of Botox diluted in 1 mL of normal saline or 1 mL of saline alone. Skin scars were assessed using the modified Stony Brook Scar Evaluation Scale (SBSES) and Manchester Scar Scale (MSS) at 1, 12, and 24 weeks postoperatively. The SBSES and MSS scores of the Botox group were significantly better than those of the control group (*p* = 0.034 and *p* = 0.039). At 24 weeks postoperatively, the SBSES and MSS scores were significantly better in the Botox group (*p* = 0.006 and *p* = 0.030). BTA injected into the incision site can suppress postoperative scar formation and thereby improve the cosmetic outcome.

## 1. Introduction

Conventional thyroid surgery using Kocher’s neck incision is the standard operative method for a variety of thyroid diseases [1]. However, the incision leaves a permanent operative scar on the neck, and the poor aesthetic result causes patients psychological distress [2]. Thyroid cancer is the most common cancer in young women [3], and hypertrophic scars develop frequently after surgery in Asian females in particular. 

The desire to improve patient quality of life through better cosmetic results after thyroid surgery has led to the use of scarless endoscopic and robot-assisted procedures [4]. However, these remote access surgeries are difficult, costly and time-consuming, such that patients in small and medium-sized hospitals, especially in developing countries, are unlikely to benefit from them. Consequently, the treatment of hypertrophic scars or keloids using pharmacological agents (such as steroids, colchicine, iron chelators, and recombinant interferon) and physical methods (such as ultrasound, cryotherapy, laser, and radiation) has been the focus of several studies [5,6]. 

Continuous physical tension on the surgical wound during the healing process prolongs inflammation and increases the risk of hypertrophic scar formation. Similarly, continuous micro-trauma due to muscle contraction in the deep layers of the skin may also lead to hypertrophic scarring or hyperpigmentation. Surgical methods aimed at reducing tension include deep muscular suturing and undermining of the skin edges, as well as making an incision along the relaxed skin tension line, but these are only partially effective [7]. A recent, alternative approach is the injection of botulinum toxin A (Botox) into the platysma muscle, which is able to reduce scar formation by minimizing the tension caused by muscle contraction. Lee et al. [8] reported the efficacy of Botox in reducing scarring in a rat surgical wound model. Botox has also been tested in clinical trials of patients undergoing cleft lip repair, and used for head and neck wound treatment [9,10,11,12]. These studies reported promising results, but they had limitations including small sample sizes, the loss to follow-up of many of the patients, and bias resulting from the inclusion of various types of wounds. 

To overcome these limitations, we conducted a prospective, double-blind, randomized clinical study to investigate the effect of Botox administration on postoperative anterior neck scarring after thyroid surgery.

## 2. Materials and Methods

### 2.1. Trial Design and Participants

This prospective, double-blind, randomized, placebo-controlled trial was conducted at a single institution. The study was approved by the Institutional Review Board of Haeundae Paik Hospital. The approval date was February 15, 2016 and the identification code was 2016-01-015.

Forty consecutive patients who underwent thyroidectomy for thyroid disease at Haeundae Paik Hospital were prospectively enrolled between January 2016 and December 2016. The inclusion criteria were (1) adult (19 years or older) patients undergoing elective surgery for thyroid disease, and (2) written informed consent provided for surgery and trial inclusion. The exclusion criteria were (1) previous history of scar problems, such as hypertrophic, inflammatory, or keloid scarring, (2) a previous cosmetic procedure on the neck that included the use of Botox, fillers, and laser treatments, (3) a history of head-and-neck surgery or radiation therapy, (4) the requirement for modified radical neck dissection, (5) previous neck surgery via an endoscopic or robotic approach, (6) current pregnancy or breast feeding, (7) allergy to Botox, (8) uncontrolled medical disease or drug abuse, and (9) refusal to participate in this trial. 

### 2.2. Randomization and Blinding

The 40 patients were randomly allocated to either the study (Botox) group or control (saline) group using a random number generator. Each random assignment was sealed individually in an opaque, sequentially numbered envelope. An independent, third-party nurse prepared a vial for each patient according to the group assignment and delivered it to the operating room at the end of surgery. The vials of the study group contained 50 U of botulinum toxin A (BTA; Nabota; Daewoong Pharmaceutical, Seoul, Korea) in 1 mL of saline, while those of the control group contained 1 mL of normal saline. Surgery was performed by two surgeons blinded to the group allocation. The same surgeons injected either BTA or placebo at the end of surgery. The randomization code was not disclosed until the end of the study. 

### 2.3. Surgery and BTA Injection

All surgeries were performed in the same manner by two endocrine surgeons (Koo DH, Bae DS). After thyroidectomy, a closed-suction drain was inserted into the operative bed from outside the wound. The midline incision between the fibers of the strap muscle was closed by continuous suturing with absorbable suture material (4–0 Vicryl). After the platysma muscle had been sutured (4–0 Vicryl) using the interrupted suture technique, a 31-gauge needle was used to inject either 50 U of Botox (study group) or the same volume of normal saline (control group). Five injections were performed at 1 cm intervals above the sutured platysma muscle (along the wound and below the incision). In all patients, the skin was closed by the interrupted suturing of one layer using absorbable suture material (6–0 Vicryl). A Steri-strip was applied to the wound for 3 weeks, with a closed dressing maintained for the first postoperative week. Patients were forbidden to apply other products aimed at improving scar appearance, e.g., silicone gel or sheets. 

### 2.4. Postoperative Follow-Up and Scar Assessment

All patients were monitored during the perioperative period for any adverse reaction to Botox. Photographs of the wound were taken at every follow-up visit by one of the authors (Cho JM), who was blinded to the group allocation. Patients were photographed using the same smartphone, in the same room, and under the same lighting conditions. The photos were then used for scar assessment on the same day by four doctors (Park JO, Cho JM, Koo DH, and Bae DS) blinded to the group allocation. The Stony Brook Scar Evaluation Scale (SBSES) and Manchester Scar Scale (MSS) were used when evaluating the photos. 

The SBSES comprises the following five items, which are assigned a score of 0 or 1 (for a total score ranging from 0 to 5): width (0 = scar width > 2 mm; 1 = scar width ≤ 2 mm), height (0 = elevated/depressed in relation to surrounding skin; 1 = flat), color (0 = darker than surrounding tissue; 1 = same color or lighter than surrounding skin), hatch marks/suture marks (0 = present; 1 = absent), and overall appearance (0 = poor; 1 = good). The MSS comprises the following five items, which are assigned scores ranging from 1–4 or 1–2 (total score of 5–18): color (1 = perfect; 2 = slight mismatch; 3 = obvious mismatch; 4 = gross mismatch), matte vs. shiny (1 = matte; 2 = shiny), contour (1 = flush with surrounding skin; 2 = slightly proud/indented; 3 = hypertrophic; 4 = keloid), distortion (1 = none; 2 = mild; 3 = moderate; 4 = severe), and texture (1 = normal; 2 = just palpable; 3 = firm; 4 = hard).

### 2.5. Statistical Analyses

Statistical analyses were conducted using SPSS for Windows software (version 18.0; SPSS, Inc., Chicago, IL, USA). A *p*-value < 0.05 was considered to indicate statistical significance. For each treatment arm, the mean change in SBSES and MSS scores from baseline to 6 months post-treatment was analyzed using a paired *t*-test and repeated measures ANOVA. The results of the study and control groups were compared using a paired Student’s *t*-test. To determine whether our sample size had sufficient statistical power, an a-priori power analysis was performed (two-sided hypothesis test; alpha level of 0.001; statistical power of 90%). Forty patients (20 in each group) were included in this study, which allowed for a 30% dropout rate. 

## 3. Results

**Figure 1:**
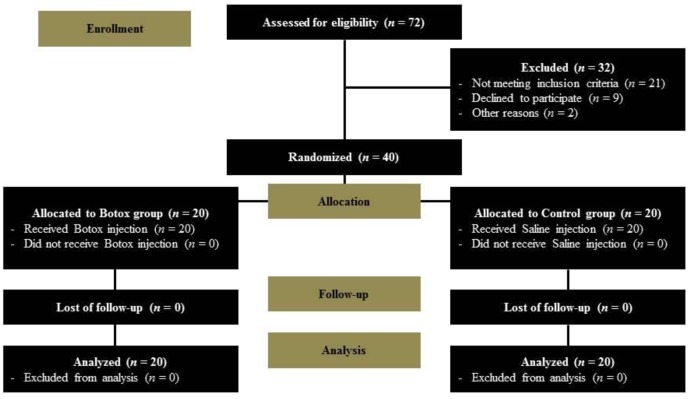
Flow chart of the clinical trial.

Of the 72 patients initially enrolled in our study, 21 did not meet the inclusion criteria, nine declined to participate, and two were excluded for other reasons. Thus, a total of 40 patients were randomly allocated to the control or study group. All 40 patients completed the study and there was no loss to follow-up (Figure 1). The demographic characteristics of both groups are summarized in Table 1. There were no significant group differences in terms of sex, general condition, tumor characteristics (size, rate of malignancy or nodal metastasis), or extent of surgery (rate of total thyroidectomy or central node dissection). The total operation time, including the duration of injection (Botox in the study group and saline in the control group), also showed no difference between the two groups. Botox-related complications (allergy, migration of the substance, or breathing issues such as wheezing and asthma) in the Botox group, and wound problems (e.g., infection or breakdown) were not observed in any patient during the follow-up period. 

**Figure 2:**
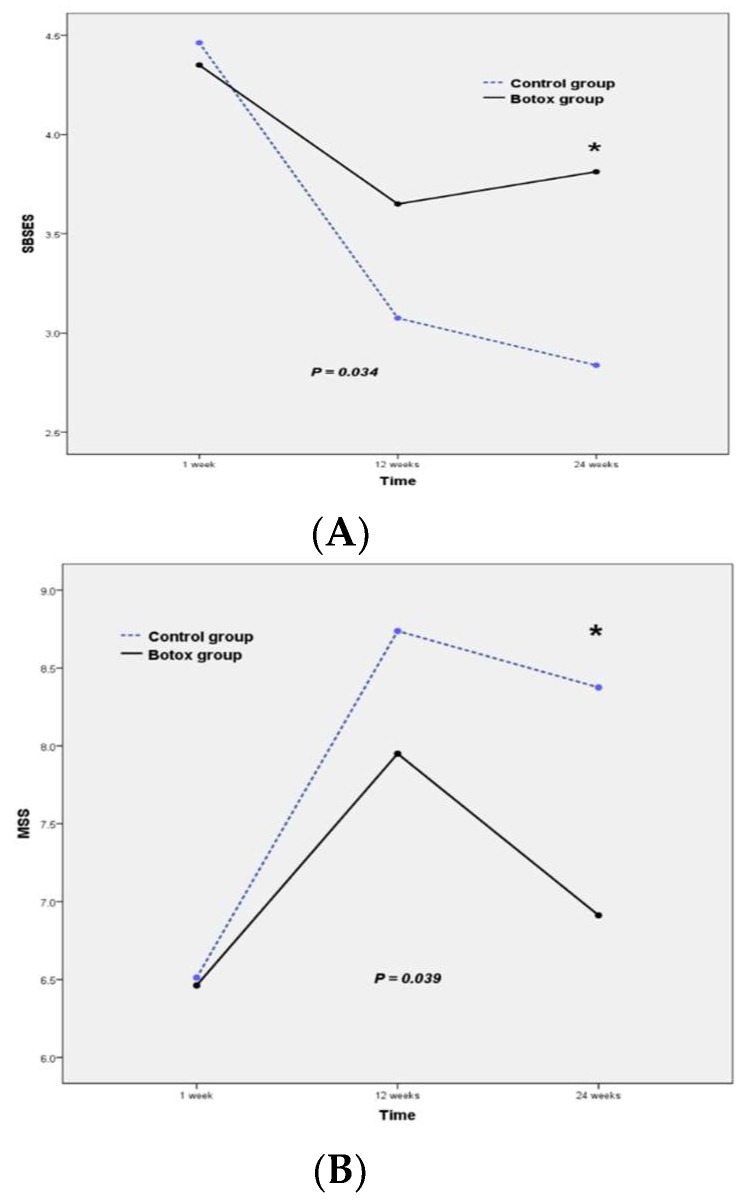
Mean score of the Botox (*n* = 20) and control groups (*n* = 20) on (**A**) the Stony Brook Scar Evaluation Scale (SBSES) and (**B**) the Manchester Scar Scale (MSS). * indicate that *p*-value is < 0.05

**Figure 3:**
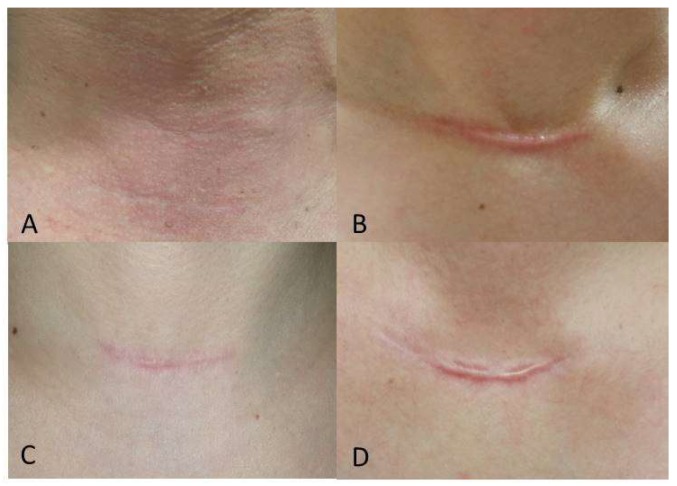
Representative postoperative scars at 24 weeks after open thyroidectomy; (**A**) A 54-year-old male in the Botox group; (**B**) a 41-year-old female in the control group; (**C**) a 46-year-old female in the Botox group; and (**D**) 54-year-old female in the control group.

The mean SBSES and MSS scores of the Botox and control groups were not significantly different at one week (*p* = 0.615 for SBSES and 0.867 for MSS) or 12 weeks (*p* = 0.085 for SBSES and 0.147 for MSS) postoperatively, but at 24 weeks postoperatively, patients in the Botox group had significantly better scores compared to those in the control group (Table 2, SBSES: *p* = 0.006 and MSS: *p* = 0.030). The repeated measures ANOVA showed significant differences in SBSES and MSS scores between the Botox and control groups (Figure 2, *p* = 0.034 and *p* = 0.039, respectively). Specifically, the SBSES score tended to be higher, and the MSS score lower, in the study group than in the control group. Figure 3 shows representative postoperative scars of patients in the Botox and control groups at six months after open thyroidectomy. The SBSES score was 5 and 4 in Botox patients A and C, and 1 and 2 in the control patients B and D, respectively. The MSS scores were 5 and 7 in Botox patients A and C, and 12 and 11 in control patients B and D, respectively. 

## 4. Discussion

A scar occurs due to the creation of new connective tissue that fills defects caused by disease or injury, as a facet of normal wound healing. The inflammatory, proliferative, and remodeling phases that make up the wound healing process protect the body from pathogens and foreign bodies. The formation of granulation tissue and extracellular matrix during the inflammatory and proliferative phases is tightly regulated. However, when the balance between production and degradation is disrupted, a hypertrophic scar or keloid is formed. Other factors leading to hypertrophic scars or keloids include excess tension on the wound, inadequate suturing, and poor nutritional status of the patient [13]. In addition, repeated micro-trauma caused by continuous displacement of the injured tissue can intensify the inflammatory response and related metabolic activity during healing, leading to the increased extracellular deposition of collagen and glycosaminoglycans and, as a consequence, a hypertrophic scar.

Botulinum toxin is a potent neurotoxin that results in flaccid paralysis of the treated muscle for up to six months. Botulinum toxin is derived from the bacterium *Clostridium botulinum* and exists in serotypes A–F. BTA and botulinum toxin B (BTB) are used in the treatment of various disorders. BTA received US Food and Drug Administration (FDA) approval for use in the treatment of neurological disorders such as focal spasticity, bladder dysfunction in adult patients, and skin and skin appendage disorders such as severe hyperhidrosis of the axillae. It is also used for temporary improvement in the appearance of moderate to severe facial lines. BTA is now used extensively in cosmetic procedures. Nabota^®^ (Daewoong Pharmaceutical), the BTA formulation tested in this study, was introduced in 2014 and is FDA-approved. It shows high efficacy and safety in the treatment of facial wrinkles and upper limb spasticity after stroke, by weakening or paralyzing the affected muscles [14,15]. 

Our prospective randomization study differed methodologically from previous studies. First, in the randomized controlled studies of Kim et al. [11] and Phillips et al. [12], the scars of thyroidectomized patients were split in two, with one half injected with Botox and the other half left untreated as the control. However, this ignored the possibility that the Botox would spread and affect the control side. Thus, in our study, the effect of Botox on scar formation was investigated in separate groups of patients. Second, in the study of Kim et al. [11], thyroidectomy patients were injected with Botox postoperatively, during a follow-up visit to the outpatient clinic, whereas in our patients BTA was injected directly into the muscle just before closure of the skin during surgery. Thus, our approach allowed for immediate and individualized assessment of the wound.

After thyroid surgery, a prominent scar is evident for 24 weeks. Although there were individual differences, the scars of patients in our control group tended to be darker, broader, higher, more hypertrophic, and firmer. While there was no significant difference in the MSS or SBSES scores (the higher the score, the better for SBSES and the lower, the better for MSS) between the study and control groups at one week or 12 weeks postoperatively, after 24 weeks the scores for the scars of the control group patients were worse than those of the study group patients, on both scales, which means that the SBSES score was high and the MSS score was low in the study group. BTA has a duration of action of up to six months, which is similar to the amount of time needed for the normal wound healing process to be completed. This implies a continuous muscle tension-alleviating effect of BTA, which would explain the superior results of the Botox group at the six-month follow-up.

This study had several limitations. First, both scar evaluation scales (SBSES, MSS) are based on subjective parameters, since a simple and objective method for scar evaluation is not yet available. Second, the number of study subjects was only 40 (treatment 20, placebo 20), which means that the conclusion may not be reliable. Therefore, although our study is a prospective double-blind randomized trial, it should be regarded as preliminary clinical research. Third, no histologically-based group comparisons could be performed, as we could not biopsy the healed scar. Thus, the beneficial effect of BTA on the wound healing mechanism itself could not be assessed pathologically. Finally, the optimal dose of BTA for wound healing has yet to be determined, thus, we used the dose recommended for cosmetic treatment of the neck. Despite these limitations, our study was a well-designed, prospective double-blind randomized trial that may encourage further investigation of the utility of BTA for treating thyroidectomy patients. Additional studies including large populations are required.

## 5. Conclusions

Intraoperatively, local application of BTA to the thyroidectomy wound may reduce hypertrophic scar formation by alleviating muscle tension, and thereby improve the cosmetic outcome. Further studies including large populations are needed to determine both the mechanism underlying the observed positive effect of BTA on cosmetic outcome in thyroidectomy patients, and the optimal dose.

## Figures and Tables

**Table 1:** Demographic data of the patients (N = 40).

Parameters	Study Group (*n* = 20)	Control Group (*n* = 20)	*p*
Age (mean ± SD) (range)	50.20 ± 9.51 (29–67)	50.50 ± 8.88 (31–67)	0.918
Sex			
Female	16 (80%)	19 (95%)	0.151
General condition			
BMI (kg/m²)	24.85 ± 3.39	24.15 ± 4.14	0.562
ASA grade	1.70 ± 0.47	1.75 ± 0.44	0.731
Tumor			
Size (cm)	0.78 ± 0.38	0.94 ± 0.77	0.384
Malignancy	19 (95%)	19 (95%)	1.000
Node metastasis	7 (35%)	3 (15%)	0.144
Surgery			
Total thyroidectomy	10 (50%)	13 (65%)	0.337
Lobectomy	10 (50%)	7 (35%)	
CND			0.549
None	0 (0%)	1 (5%)	
Unilateral	17 (85%)	17 (85%)	
Bilateral	3 (15%)	2 (10%)	
Operation time (min)	109.25 ± 25.14	114.75 ± 26.87	0.508
Amount of bleeding (ml)	76.05 ± 40.91	56.75 ± 32.69	0.108

SD, standard deviation; BMI, body mass index; ASA, American Society of Anesthesiologists; DM, diabetes mellitus; CND, central node dissection.

**Table 2:** Mean and standard deviation Stony Brook Scar Evaluation Scale (SBSES) and Manchester Scar Scale (MSS) scores of the study (Botox) and control (saline) groups (N = 40).

Parameter		Study Group (*n* = 20)	Control Group (*n* = 20)	*p*
SBSES	1 week	4.35 ± 0.86	4.46 ± 0.50	0.615
	12 weeks	3.65 ± 1.09	3.07 ± 0.96	0.085
	24 weeks	3.81 ± 1.14	2.84 ± 0.99	0.006
MSS	1 week	6.46 ± 0.96	6.51 ± 0.92	0.867
	12 weeks	7.95 ± 1.48	8.74 ± 1.86	0.147
	24 weeks	6.91 ± 1.59	8.38 ± 2.41	0.030
